# Precious Property or Magnificent Money? How Money Salience but Not Temperature Priming Affects First-Offer Anchors in Economic Transactions

**DOI:** 10.3389/fpsyg.2018.01099

**Published:** 2018-07-04

**Authors:** Yannik M. Leusch, David D. Loschelder, Frédéric Basso

**Affiliations:** ^1^Department of Psychological and Behavioural Science, London School of Economics and Political Science, London, United Kingdom; ^2^Institute of Management and Organization, Leuphana University of Lüneburg, Lüneburg, Germany

**Keywords:** anchoring, loss aversion, resource framing, money, embodiment, temperature priming, bartering

## Abstract

The present study aims for a better understanding of how individuals’ behavior in monetary price negotiations differs from their behavior in bartering situations. Two contrasting hypotheses were derived from endowment theory and current negotiation research to examine whether negotiators are more susceptible to anchoring in price negotiations versus in bartering transactions. In addition, past research found that cues of coldness enhance cognitive control and reduce anchoring effects. We attempted to replicate these coldness findings for price anchors in a distributive negotiations scenario and to illuminate the potential interplay of coldness priming with a price versus bartering manipulation. Participants (*N* = 219) were recruited for a 2 × 2 between-subjects negotiation experiment manipulating (1) monetary focus and (2) temperature priming. Our data show a higher anchoring susceptibility in price negotiations than in bartering transactions. Despite a successful priming manipulation check, coldness priming did not affect participants’ anchoring susceptibility (nor interact with the price/bartering manipulation). Our findings improve our theoretical understanding of how the focus on negotiation resources frames economic transactions as either unidirectional or bidirectional, and how this focus shapes parties’ susceptibility to the anchoring bias and negotiation behavior. Implications for theory and practice are discussed.

## Introduction

Venezuela currently faces one of the worst economic crisis in decades (Cerra, 2017). In times like these, trust in a currency can drastically drop to the point of hyperinflation. As a consequence, in Caracas – Venezuela’s capital – people have started to barter and trade their goods over Facebook, Instagram and WhatsApp. A kilo of pasta is exchanged for a packet of diapers (Fishwick, 2016). Money is not regarded as a reliable store of value anymore, therefore economic exchange occurs via bartering. But how do the outcomes from bartering negotiations differ from typical deals that involve money? The present research examines how economic behavior is affected by the characteristics of resources around which an economic transaction can revolve (i.e., money in price negotiation versus commodities in bartering).

To elucidate this research question, we opted to focus on a well-established, robust, and highly influential effect in negotiations—the anchoring effect of first offers (see Klein et al., 2014). First-offer anchors have been investigated in multiple studies and have been shown to strongly impact behavior and negotiation outcomes (Galinsky and Mussweiler, 2001; Loschelder et al., 2016). Here, we specifically aim to explore how the focus on different resources – money versus commodities – affect the potency of anchors in price negotiations and bartering transactions, respectively. Interestingly, the current literature yields two plausible but *opposite* hypotheses for how monetary resources and commodity resources influence economic decisions and negotiators’ anchoring susceptibility. On one hand, negotiation research suggests that being in possession of money typically leads to a predominant focus on one’s own resource and could lead to a weaker anchoring effect in the price negotiation (vs. bartering) because people are averse to spend their own money (Neale et al., 1987; Appelt et al., 2009). On the other hand, building on previous conceptualizations of the endowment effect (Thaler, 1980; Kahneman et al., 1990), one could predict that people possessing a tangible object (rather than money) are more concession averse and would hence be less susceptible to be anchored in bartering situations (vs. price negotiation). To examine these competing predictions, we asked participants to engage in either a price negotiation or a barter transaction, allowing them to invest either their money or their own tangible resource. Hereby, we did not manipulate the role of the participants as either buyers or sellers, but instead sought to directly compare the effects of anchoring susceptibility in bartering versus price negotiation scenarios.

To investigate the psychological influence of monetary resources in negotiations more closely, we also tried to shed light on the relationship of physical coldness and money perception: past research has established a link between money and coldness perception. Here, we also examined the reversed link of coldness priming on monetary perception (and a potential interaction with our price negotiation vs. bartering manipulation; Reutner et al., 2015). Since this relationship has up-to-date only been found in non-economic situations, we tried to examine whether this potential link is generalizable to economic transaction scenarios, such as negotiations. Hereby, we sought to determine the extent to which perceptions of coldness potentially interact with the focus on money versus tangible goods. Before detailing these manipulations and reporting our experimental study, we will briefly review relevant research on first-offer anchoring, money and the endowment effect, as well as work on coldness and embodiment.

## First Offers as Anchors

As a classic judgmental bias, anchoring constitutes the assimilation of judgment to a relevant or irrelevant numeric standard (Tversky and Kahneman, 1974; Mussweiler and Strack, 2000a). Anchoring is considered a highly robust phenomenon (Klein et al., 2014) as anchors provide orientation for judges’ decisions in situations of uncertainty. In negotiations, recipients are anchored by the first offer and assimilate their counteroffer to this numeric value. Ultimately, final agreement gravitates toward the first-offer anchor and recipients often secure lower individual profits than first moving senders (Loschelder et al., 2014b, 2016). To account for the impact of anchoring on negotiation, two influential mechanisms have found empirical support: insufficient adjustment and selective accessibility. First, negotiators mentally adjust their counteroffers away from a first-offer anchor (Tversky and Kahneman, 1974; Epley and Gilovich, 2001, 2004, 2006). In a negotiation, parties trade concessions and eventually meet somewhere in the middle of first offer and counteroffer. When a counteroffer is insufficiently adjusted, final outcomes gravitate toward the anchor established through the sender’s opening proposal (see Epley and Gilovich, 2004; Loschelder et al., 2017). Second, anchors can render information selectively more accessible that supports rather than refutes an anchor value. For instance, negotiators would primarily focus on information that is consistent with a first-offer anchor. High anchors, such as a higher price for a car, activate positive attributes (e.g., valuable equipment and low mileage), and this anchor-consistent knowledge lends support to the anchor and bolsters its potency (Mussweiler and Strack, 2000a,b). In sum, abundant research has shown that first offers function as anchors and attribute a bargaining advantage to the first-moving party: an increasing anchor extremity leads recipients to insufficiently adjust their counteroffers and activates knowledge supporting the anchor. In light of the plethora of research supporting the robust and dominant impact of first-offer anchors, the astute reader may wonder how negotiators can counteract this first-offer anchoring bias. In the present paper, we will examine both a relative focus on money versus tangible resources and a coldness priming as potential moderating factors for the ever-so-strong anchoring impact.

## Magnificent Money vs. Precious Property?

Competing predictions can be derived from the literature concerning whether anchoring susceptibility is more pronounced in price negotiations (money for item) versus in bartering negotiations (item for item).

### Money Hypothesis

Many studies have emphasized the role of money as a multifunctional or ‘all-purpose social resource’ that can be used in exchange for many goods and is linked to numerous key psychological processes. For instance, it has been shown that its relevance is strong enough to compensate for human needs such as self-esteem or self-sufficiency (Vohs et al., 2006; Zhang, 2009). The psychological power of money clearly goes beyond its multi-functionality as it has, for instance, been found to have drug-like effects on people (Lea and Webley, 2006). In light of money’s influential role in our society, it is not surprising that money is often viewed as the central point of focus in negotiations. Neale et al. (1987) have shown that parties in possession of money feel more powerful and less obligated to close the deal than their counterparts. With this leverage, and focusing on their own money, *buyers* have often been shown to attain better outcomes in economic exchanges than sellers (Neale et al., 1987; Trötschel et al., 2015a). Money’s dominant role in negotiation is, at least in part, supported by its characteristic of being highly divisible (Appelt et al., 2009). A large sum of money can be easily divided into smaller elements. Concessions can be made much more easily. Buyers can simply offer 350€ instead of only 320€ for a bike. It follows from this divisibility characteristic that most negotiations are perceived as a price-negotiation rather than a commodity negotiation (Trötschel et al., 2015b). When no resource is accentuated through the framing of a negotiation proposal (e.g., “offer x for y” versus “request y for x”; see Trötschel et al., 2015b), the dominant role of the highly divisible resource money makes both parties focus on the monetary resource. Therefore, buyers seek to spend as little money as possible; sellers, on the other hand, seek to maximize their profit. Buyers are placed in a loss frame, sellers in a gain frame (Carmon and Ariely, 2001). Previous research has shown that parties acting in a loss frame have a higher resistance to concede (Kahneman, 1992; DeDreu et al., 1995). If a party has a high concession aversion they are more likely to receive a higher outcome (DeDreu et al., 1995). This is why buyers in price negotiations appear to often receive the higher outcomes than sellers (McAlister et al., 1986). It follows from this research that participants in possession of money should be focused on losing their own resource and thus should be more concession averse and less prone to be anchored than participants in a bartering transaction (H1a).

### Endowment Hypothesis

In contrast to this literature on money and the buyer-advantages in negotiations (Trötschel et al., 2015a,b), the endowment effect sheds a different light on buyer-seller interactions. The endowment effect is the tendency for sellers to ascribe a higher value to items they possess than buyers are willing to pay for it. It has been examined numerous times in behavioral economic research (Kahneman et al., 1990; Heffetz and List, 2014; Sprenger, 2015). In line with the prospect theory, the endowment effect can be explained via a shift of reference points for sellers toward their own commodity resource, thus emphasizing their own losses more than their own potential gains (Kahneman and Tversky, 1979). Conversely, on the buyers’ side, the loss of their own money will loom larger than the gain of an object they do not possess. This has implications for economic exchanges as in the case of negotiations. For instance, as shown by Majer et al. (2018), the focus on one resource triggered by procedural framing – for instance: “I offer my X for your Y” versus “I request your Y for my X” – can lead to a lower anchor susceptibility in negotiators. It follows from this literature and these findings that, in an economic transaction, parties in possession of a commodity may experience an endowment effect and thus should be more concession averse and less prone to be anchored than participants in a price negotiation (H1b).

In the present paper, we examined these two competing predictions and we tested whether being in possession of money reduced anchoring susceptibility when compared with being in the possession of an item in a bartering negotiation (H1a vs. H1b). To address this research question, we manipulated whether actors in an economic exchange transaction would either barter commodities or invest money. To avoid potential confounds, both experimental conditions realized identical numeric values, quantities, and ultimately an identically divisible resource (see details below).

## Embodiment and Coldness Priming

Surprisingly little was known about the psychology of money, so that it has been called “one of the most neglected topics in the whole discipline of psychology” (Furnham and Argyle, 1998, p.2). However, in the past two decades psychological research has started to shed light on this under-investigated area (see Vohs et al., 2006). An important insight from this research is that money as a concept may be closely linked to feelings of coldness (Reutner et al., 2015). This money and coldness link led us to additionally examine the potential moderating impact of coldness priming on negotiators’ anchoring susceptibility. As first shown by Williams and Bargh (2008), temperature stimuli can change behavior and influence interpersonal judgement. For instance, people primed with coldness are more likely to choose a gift for themselves than for others. Many subsequent studies then focused on the connection of physical and social warmth, and provided hints for its bi-directionality (IJzerman and Semin, 2009; Hu et al., 2016), while more recent studies have also taken coldness (rather than warmth) into consideration (Nakamura et al., 2014; Willemse et al., 2015; Zhong and Leonardelli, 2015). The expressions ‘*keeping it cool’* or ‘*playing it cool’* are used in our everyday life to express self-control and non-emotional behavior. The adjective ‘cool’ to describe a person is not necessarily related to social disconnection, but is in its core a description of a person able to remain calm and controlled in the presence of emotional or arousing circumstances. While warmth is linked to emotions, everyday language links coldness metaphors to describe *rational* behavior. In line with this observation, coldness-primes seem to have a positive impact on cognitive self-control (Halali et al., 2016). Sassenrath et al. (2013) recently found that physical coldness enhances peoples’ perspective-taking performance: the egocentric perspective was reduced when subjects were exposed to cold temperature cues. However, it remains unanswered from prior research whether such enhanced cognitive control through primed coldness might also reduce the potency of (first-offer) anchors. We propose that, if coldness priming does indeed enhance cognitive control, it should lead to an overall better adjustment away from anchors before the negotiation, resulting in a reduced anchoring susceptibility (coldness main effect; H2).

In contrast to this coldness main effect, one can also derive a moderation hypothesis from the literature. Reutner et al. (2015) showed in their experiments that symbolic reminders of money can cause a lower estimation of the room temperature, thus establishing an explicit connection between money and the warm/cold dimension. Past research has only shown the unidirectional influence of money on sensations of (cold) temperature. However, many other conceptual relationships that are grounded in embodied cognition appear to be bi-directional, as for example social and physical warmth (Williams and Bargh, 2008; IJzerman et al., 2012). Therefore, based on the assumption that the link of money and coldness might also be of such bi-directional nature, we empirically tested the prediction that physical coldness affects individuals’ perception of money (and makes negotiators less willing to spend it). We hypothesized that coldness priming would exacerbate the concession aversion in monetary (vs. bartering) negotiations and thus further counteract the anchoring impact of first offers, again resulting in a reduced anchoring susceptibility (moderation hypothesis H3).

## Materials and Methods

### Participants and Study Design

A statistical power analysis to determine the necessary sample size was conducted using G^∗^Power (Faul et al., 2007). Given that the effect sizes found in the relevant literatures on (1) procedural framing that examines different accentuations of resources (average $\eta_{\text{p}}^{2}$ = 0.25; Trötschel et al., 2015b), and on (2) the money-coldness link (Reutner et al., 2015; $\eta_{\text{p}}^{2}$ = 0.19) seemed relatively large and potentially larger than the true population effects, we opted for a more conservative effect size estimate of $\eta_{\text{p}}^{2}$ = 0.06 (*d* = 0.5)—a moderate effect according to Cohen’s conventions. The power and alpha-error level were set to 1-β = 0.90 and α = 0.05 (Bortz and Döring, 2006). To detect a possible significance in an ANOVA for main and interaction effects, G^∗^Power indicated a minimal sample size of 171 participants. Anticipating that not all participants would meet the experimental standards and would have to be excluded based on pre-defined exclusion criteria (e.g., reported knowledge of the anchoring effect), data collection was terminated after all signed up participants (*N* = 219) had participated in the study. We did not examine any data prior to termination of data collection. The final sample included 189 subjects (*M*_age_ = 23.08, *SD* = 4.55) because the remaining participants indicated suspicion of the priming manipulation (*n* = 14) or spent too much time between the priming task and the negotiation (more than 60 s; *n* = 16). Both of these criteria were defined prior to data collection.

A 2 × 2 factorial between-subjects design with temperature priming (cold vs. warm) and resource focus (money vs. commodity) was realized.

### Experimental Manipulation

All participants were randomly assigned to an experimental condition. Participants engaged in an economic transaction framed as either a price negotiation (money focus) or a bartering negotiation (commodity focus). Both conditions were realized as a computer-simulated distributive transactions, in which participants acquired a bike stand (either by investing money or by bartering for it; details below).

Participants were primed with either cold or warm temperature. In the cold priming group, participants were asked to read a newspaper article on a marathon taking place in an arctic setting (cold priming group) versus on a Spanish vacation island (warm priming group). A similar, successful priming of coldness has been conducted by Halali et al. (2016), when they presented an experimental task in front of a wintery landscape background to prime coldness. In addition to a similar picture in the newspaper article, we reinforced this prime with the use of pictorial language to describe the arctic or summery scenery. This description was presented from a first-person point of view from one of the marathon runners. Recent research suggests stronger embodiment effects when stimuli are presented from a first person perspective (Macrae et al., 2013). In line with the moderation hypothesis outlined above, we reasoned that a successful manipulation of the coldness priming would result in different foci on the resources, with coldness-primed subjects focusing more on their own resource (especially their own money) than subjects exposed to warmth-related stimuli.

In addition, we manipulated the negotiations to center around money (price negotiation) versus the exchange of commodities (bartering negotiation). In the money condition, participants were instructed to invest their money (between 1€ and 440€), whereas in the bartering condition, we deliberately refrained from mentioning money but instructed participants to engage in an exchange transaction trading their own resource (between 1 and 440 bike bells) for the bike stand. To ensure that the characteristics of resources were equal and thus had no unintended (numeric) influence on the negotiation outcome, the two conditions – bartering resources and spending money – were identical in objective value and bike bells were said to be worth 1€ each. Importantly, divisibility for both conditions was identical, allowing for concessions in full units (1–440) and profit rates were also identical (participants were told that after the negotiation all remaining items could be sold for 1€ each with no additional costs). In other words, the monetary and non-monetary conditions had the same properties, except from their explicit nature of being money in a price negotiation versus a commodity in a bartering negotiation.

### Negotiation Task

Participants were presented with written instructions to the following economic transaction scenario. Participants were asked to imagine being the owner of a small bike-repair shop. In both experimental conditions, they were to acquire a used work stand for repairing bikes (i.e., a non-divisible commodity item) from another bicycle store in town. In line with past research (e.g., Galinsky and Mussweiler, 2001), the experiment provided participants with uncertain information and a potential range in which the value of the bike stand could fall. Participants in the non-monetary bartering condition were additionally introduced to the fact that their bicycle shop had bike bells in stock and that the owner of the other bike shop had accepted to barter bells for his stand. Hence, whereas in the price condition, negotiators invested their own money for the bike stand (1€–440€), participants in the bartering condition traded between 1 and 440 bike bells for the same stand.

### Dependent Variables

As dependent variables, we measured participants’ first and second counteroffer and their willingness to pay (WTP), as well as the concession range (difference between first counteroffer and WTP). To check whether the coldness manipulation did indeed result in a stronger focus on participants’ own resources, we asked participants which resource they had predominantly focused on during the economic transaction (1 = *own resource* to 7 = *other’s resource*). To illuminate the underlying psychological mechanism, we assessed participants’ experienced concession aversion (i.e., “I had a hard time making concessions toward my negotiation opponent,” “I did not like to comply with the seller with my money/commodity” [reverse coded]; *r* = 0.552, *p* < 0.001).

### Procedure

The transaction started with a first offer from participants’ (simulated) negotiation opponent. This first-offer anchor was 500€ or 500 bells, respectively. The anchor was set above the highest possible price that the participants could afford to prevent people from accepting the first request right away. All proposals throughout the study were phrased as neutral as possible to avoid unintentionally highlighting either of the resources (“The seller starts with a *suggestion* of 500× in exchange for the bike-stand; Trötschel et al., 2015b). Over two negotiation rounds the concession aversion was measured (both counteroffers were rejected by the opposing seller); participants were finally asked to name their highest WTP (maximum number of € or bells they were willing to invest for the bike stand). Subsequently, participants were informed that their WTP had been accepted by the seller and that the negotiation was closed. All participants were debriefed and thanked for their participation.

## Results

### Manipulation Check

Indicating a successful coldness priming manipulation and in line with our predictions, participants in the cold condition reported a stronger focus on their own resources (*M* = 2.67, *SD* = 1.36) than participants exposed to warmth stimuli (*M* = 3.06, *SD* = 1.54), *t*(187) = −1.90, *p* = 0.03, *d* = 0.28 (one-tailed). Even though the effect-size turned out smaller than expected, it is consistent with what has been reported in the literature (Steinmetz and Posten, 2017, Study 1b), and a successful manipulation can be assumed.

### Anchoring Susceptibility

In line with the endowment hypothesis (H1b), participants in the commodity condition were anchored less—they made lower, thus more self-serving, counteroffers (*M* = 208.92, *SD* = 59.01) than participants in the money condition (*M* = 227.58, *SD* = 59.17). The two-factorial ANOVA for *first counteroffer* revealed a main effect for resource *F*(1,185) = 4.70, *p* = 0.031, $\eta_{\text{p}}^{2}$ = 0.025. Neither the main effect for coldness priming (*F*[1,185] = 0.069, *p* = 0.793; H2), nor the interaction effect of Coldness × Money Salience were significant, *F*(1,185) = 1.74, *p* = 0.188. Hence, contrary to the moderation hypothesis (H3), participants exposed to coldness did not display a lower anchor susceptibility when negotiating about money rather than bartering their commodities.

### Negotiation Process

**FIGURE 1:**
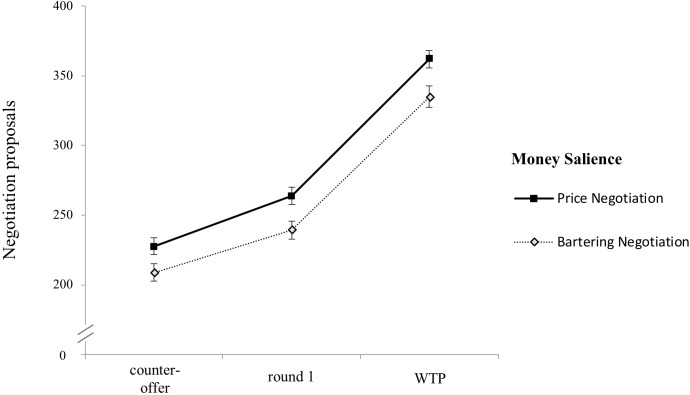
Participants’ negotiation proposals as a function of negotiation round and money salience. Participants in the bartering negotiation (gray circles) were anchored less by the opponents’ first offer than participants in the price negotiation (black squares). This initial difference for counteroffers was maintained over the course of negotiations and culminated in a lower willingness to pay (WTP) for the bartering compared to the money condition. Error bars represent +/– 1 *SEM*.

To assess possible differences between experimental conditions over the course of the negotiation, we performed a 2 (Coldness Prime) × 2 (Money Salience) × 3 (Negotiation Round) ANOVA with repeated measures for the latter Round factor. As expected, a main effect for Round occurred—all participants conceded over the course of the negotiation, *F*(2,370) = 656.70, *p* < 0.001 $\eta_{\text{p}}^{2}$ = 0.78 (**Figure 1**). All interaction effects were non-significant—both two-way (all *F*s < 0.741, *p*s > 0.477), and three-way (*F* = 1.31, *p* = 0.272). However, in line with the endowment hypothesis, a strong main effect again emerged for the money salience manipulation, *F*(1,185) = 8.63, *p* = 0.004, $\eta_{\text{p}}^{2}$ = 0.045 (H1b).

Across the negotiation rounds, participants in the bartering negotiation were anchored less – made lower and more self-serving proposals – than participants in the price condition (**Figure 1**). In summary, the reduced anchor susceptibility in first counteroffers was maintained over the negotiation process and culminated in a lower WTP.

### Robustness Check

The high power and the large sample size of the present study allowed us to alleviate potential concerns about a type-I error by conducting a robustness check that replicated the reported main effects for money vs. bartering in two independent subsamples. Specifically, we randomly generated two subsamples of completely independent participants. For both of these subsamples, we then re-ran the 2 (Coldness Prime) × 2 (Money Salience) × 3 (Negotiation Round) ANOVA with repeated measures for the latter Round factor. As expected, a significant main effect emerged for Money Salience in both the first subsample, *F*(1,89) = 7.38, *p* = 0.008, $\eta_{\text{p}}^{2}$ = 0.076, and the second subsample, *F*(1,92) = 4.86, *p* = 0.030, $\eta_{\text{p}}^{2}$ = 0.05.

In short, both randomly generated subsamples independently corroborate the analyses reported above and thus markedly attenuate the likelihood of a type-I error to a total of 0.25% (i.e., 0.05 ^∗^ 0.05 = 0.0025).

### Underlying Mechanisms

**FIGURE 2:**
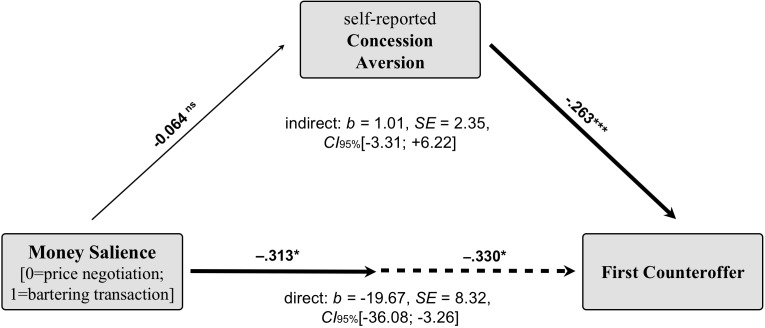
Mediation analysis: the direct effect of money salience on first counteroffer was significant, the indirect effect through participants’ concession aversion was not due to a non-significant a-path. Path coefficients are unstandardized regression weights for *Z*-scored mediator and dependent variable.

We next examined how (1) participants’ self-reported concession aversion correlated with their anchoring susceptibility, and (2) how our coldness priming and money salience manipulations affected this psychological mechanism. First, higher levels of concession aversion were, as expected, correlated with lower counteroffers (*r* = −0.258, *p* < 0.001) and a lower WTP (*r* = −0.246, *p* < 0.001). Second, contrary to our predictions, a 2 (Coldness Prime) × 2 (Money Salience) ANOVA showed no systematic impact on participants’ concession aversion (all *F*s < 0.63, *p*s > 0.428). Accordingly, mediation analyses using the Process macro (Hayes, 2017, model 4; **Figure 2**) did not show that the direct effect of money salience (0 = price negotiation; 1 = bartering transaction) on first counteroffers [*B =* −19.67, *SE* = 8.32, *CI*_95%_(−36.08; −3.26)] was mediated through an indirect effect of self-reported concession aversion [*B =* 1.01, *SE* = 2.35, *CI*_95%_(−3.31; 6.22)].

## Discussion

The present paper examined whether (1) temperature priming and (2) an economic transaction framing (monetary versus commodity focus) impact negotiators’ anchoring susceptibility and ultimately their willingness to pay. We empirically examined two competing predictions for the price versus bartering manipulation, as well as a potential interaction of coldness priming with a monetary focus. Our results are much in line with the endowment hypothesis according to which parting with one’s tangible items in a bartering negotiation leads to a reduced anchoring susceptibility compared to investing one’s money in a price negotiation. In other words, our results show that participants who bartered their own resources were anchored much less by the opponent’s first offer than participants who invested money in a price negotiation. In fact, this pattern unfolded over the entire course of the negotiation, during which participants in possession of the commodity made lower offers and ultimately showed a lower willingness to pay. As illustrated in **Figure 1**, our results show that the interaction of negotiation process and price/bartering conditions was non-significant, which means that parties in the money and in the commodity condition made equally large concessions from their first counteroffer to their final WTP. Ultimately, this indicates that – due to their lower first counteroffer – participants in the commodity condition were not anchored as much as the ones in the monetary condition.

### Limitations and Future Research

More closely inspecting the nature of price negotiation versus bartering transactions may shed novel light on the observed outcome pattern, some limitations, and avenues for future research. In our everyday lives, we use money in numerous situations, as cold hard cash at the coffee place or as quick digital payment at the gas station. Most of the time, we consider these transactions as unidirectional: we do not necessarily frame it as selling our money but rather see it as the purchase of coffee or gasoline. In the situation of a barter deal, people may perceive a highly similar (almost identical) transaction as more bi-directional. Hence, the exchange of resources in a bartering transaction is more symmetrical in that both negotiators part with their own belonging and receive the others’ belonging accordingly. Psychologically trading one’s good may subjectively come at a greater cost—we feel concession averse to part with our precious property. Ultimately, an elevated concession aversion in a bartering transaction may lead to a lower anchoring potency of the first offer in the commodity (than in the price) condition and culminates in a lower WTP.

The present data come with the limitation that asking participants to self-report their concession aversion did not show the expected, systematic difference between prices versus bartering negotiations. This lack of systematic difference in self-reports may well be due to a lack of introspective insight of participants in terms of their perceived concession aversion, likely because a sense of loss aversion is typically not something that people are capable of observing in themselves, let alone expressing verbally when asked about it (see Nisbett and Bellows, 1977). Some of our own related work in this field also indicates that concession aversion – as a construct – is not introspectively accessible to our recruited participants (Trötschel et al., 2015a). To overcome this shortcoming, future research may additionally capture concession aversion and loss aversion not via self-reports but via more unobtrusive, direct measures—for instance, via participants’ electro-dermal activity (EDA). EDA has been shown to be a rather sensitive measure for participants’ impression of making monetary losses (e.g., Bechara et al., 1997), even when participants are not yet consciously aware of the impending losses. In the present study we followed recent research on the anchoring effect and treated a behavioral measure (anchoring susceptibility) as a downstream consequence of an underlying psychological process (loss aversion; see also Epley and Gilovich, 2001, 2006, 2010; Mussweiler and Strack, 2001, for similar approaches). Note for instance, that the prominent perception is that insufficient adjustment accounts for the anchoring effect (e.g., Epley and Gilovich, 2010), yet the adjustment process itself is difficult to measure (introspectively); it has thus been inferred from a stronger (vs. less pronounced) anchoring effect. In this regard, the present study is unfortunately no exception in that we did not find mediation evidence for subjectively experienced concession aversion—in spite of clear differences for anchoring susceptibility. As mentioned above, follow-up studies could assess the underlying psychological constructs (concession aversion, insufficient adjustment, etc.) more elaborately (and potentially with indirect, physiological measures), when comparing bartering and price negotiations. To additionally enhance a more distinct and valid psychometrical assessment of concession aversion, future research might also include an additional, validated behavioral measurement of concession aversion, such as an Ultimatum or Dictator Game. Finally, another possible explanation for why no difference in self-reported concession aversion occurred is that the characteristics of the resources may not have affected concession aversion in the negotiation process but solely negotiators’ initial anchoring susceptibility. This explanation is in line with the finding that the two groups made a comparable amount of concessions during the course of the negotiation; yet, at the same time, they attained significantly different negotiation outcomes that were due to different initial counteroffers.

In light of the present focus on negotiating buyers, we wish to note that our study sought to compare how anchoring susceptibility differs between bartering and price negotiations, not how it differs between buyers and sellers. Given that a number of studies have found highly comparable anchoring effects for both buyers and sellers (e.g., Galinsky and Mussweiler, 2001; Loschelder et al., 2014a, 2016), it seems reasonable to assume that the present findings will also extend to the seller role. In any case, we hope that our findings provide motivation for future research to pay closer attention to the extent to which negotiators’ loss aversion is affected when characteristics of the seller’s resource change—that is, when sellers receive either money or commodities in equal value for their own resource. Following our current findings, we would predict that due to higher anchoring susceptibility the first mover advantage is stronger in monetary negotiations.

Contrary to the temperature priming hypothesis and to prior substantiations of the coldness effect, we did not find evidence that coldness impacted participants’ economic decision-making or their anchoring susceptibility. We were not able to extend previous findings from Sassenrath et al. (2013) to our application in first-offer anchoring. It appears that coldness priming does not counteract anchoring effects or interact with price versus bartering manipulations in economic transaction. One might criticize that the coldness priming was unsuccessful. However, our findings indicate a successful manipulation of coldness priming in that, in line with our expectations (and past research in this field), participants in the cold condition focused more on their own resource than participants in the warm condition (see IJzerman et al., 2013). Nonetheless, the proposed interaction effect of coldness and price/bartering did not show significance. Participants in the cold/money condition did not adjust particularly farther away from the anchor compared to participants in the other three conditions. A *post hoc* power-analysis indicated that our study had a 90.32% chance to detect a directional effect of coldness priming that was at least *d* = 0.43 in size. We used this *d* = 0.43 estimate based on a meta-analysis of 322 meta-analyses in social psychology (Richard et al., 2003). Admittedly, this estimate may be over-estimating the true effect size within social psychology. Hence, for a small-to-moderate effect size estimate of *d* = 0.35 (according to common conventions by Cohen), our study sample still had a 77.4% chance of detecting an effect. It did not. Nonetheless, we cannot rule out that an even smaller effect size (e.g., *d* = 0.20) could eventually become detectable with an even larger sample size. For future research, it seems fair to point out, however, that the necessary sample size to detect a small population effect of *d* = 0.20 with an 80% power is *N* = 620 participants. This sample size unfortunately exceeds our present resources and it also raises the question whether effects of this (small) size would still be interesting and psychologically meaningful.

Ultimately, there may be many reasons why we did not replicate the effect of temperature priming. We cannot exclude that the augmented presence of stimuli through symbols (offline embodiment) has lower effects on cognition than their actual physical presence (online embodiment) (Niedenthal et al., 2005), especially in social priming experiments where effects are volatile and small (Lynott et al., 2014). In line with this argument, the only study that has provided evidence for the link between coldness and money – to our knowledge – used real physical stimuli such as hard cash money (Reutner et al., 2015); in the present study, we used symbolic/virtual money and offline cold/warm manipulations. Future research may want to contrast physical money and physical coldness perceptions with virtual money and imagined coldness to further illuminate the effect and the boundary conditions for the priming effectiveness. Future research may also examine whether actively simulating cold/warmth has a higher impact on participants anchoring susceptibility and economic decision-making than priming these visceral states (Steinmetz and Posten, 2017). For instance, instead of merely reading a newspaper article on a marathon taking place in a cold versus warm environment (priming), participants could be asked to imagine themselves for 30 s in this environment (simulation). Again, we can ascertain for the present data that, despite a successful manipulation check and adequate power to detect a population effect of small-to-moderate size, coldness did not impact negotiators’ anchoring susceptibility in price or bartering negotiations. However, our findings do not contradict previous findings on the effects of temperature priming. Follow-up studies might continue exploring the boundary conditions of temperature priming in other study designs and economic scenarios.

### Concluding Remarks

The present paper contrasted how the framing of an economic transaction as focusing on negotiators’ money versus commodity impacts their susceptibility to be anchored by the opponent’s first offer. Put simply, we contrasted the impact of magnificent money with one’s precious property and, in line with the endowment hypothesis, we found that first-offer anchors were less potent in bartering transactions.

## Ethics Statement

This study was carried out in accordance with the recommendations of the Ethics Committee for psychological research of Leuphana University. Written informed consent was received from all subjects in accordance with the Declaration of Helsinki. The conducted study involved no deception of participants and falls under the general approval of the Ethics Committee for low-risk level experiments.

## Author Contributions

YL, DL, and FB contributed to the conception and design of the study. YL conducted the experiment, the statistical analyses, and drafted a first version of the paper to which DL and FB provided several revisions. All authors contributed to the paper and approved it for publication.

## Conflict of Interest Statement

The authors declare that the research was conducted in the absence of any commercial or financial relationships that could be construed as a potential conflict of interest.
